# Familial Pancreatic Cancer

**DOI:** 10.3390/cancers2041861

**Published:** 2010-11-10

**Authors:** Henry T. Lynch, Jane F. Lynch, Stephen J. Lanspa

**Affiliations:** 1 Department of Preventive Medicine and Public Health, Creighton University, 2500 California Plaza, Omaha NE 68178, USA; E-Mail: jalynch@creighton.edu (J.L.); 2 Department of Gastroenterology, Creighton University, 2500 California Plaza, Omaha NE 68178, USA; E-Mail: stephenlanspa@creighton.edu

**Keywords:** phenotypic and genotypic heterogeneity, high mortality, genetic counseling, biomarker paucity, FAMMM syndrome, Li-Fraumeni syndrome, Lynch syndrome, pancreatic cancer

## Abstract

Pancreatic cancer’s high mortality rate equates closely with its incidence, thereby showing the need for development of biomarkers of its increased risk and a better understanding of its genetics, so that high-risk patients can be better targeted for screening and early potential lifesaving diagnosis. Its phenotypic and genotypic heterogeneity is extensive and requires careful scrutiny of its pattern of cancer associations, such as malignant melanoma associated with pancreatic cancer, in the familial atypical multiple mole melanoma syndrome, due to the *CDKN2A* germline mutation. This review is designed to depict several of the hereditary pancreatic cancer syndromes with particular attention given to the clinical application of this knowledge into improved control of pancreatic cancer.

## 1. Introduction

It is estimated that in the United States during 2009, 21,050 males and 21,420 females will be diagnosed with pancreatic cancer (PC), giving a total of 42,470. Its mortality is estimated at 18,030 men and 17,210 women giving a total of 35,240. Its mortality closely approximates its incidence [1], thereby indicating its dismal outlook [2]. As clinician-scientists, we are often faced with the patient’s emotionally steeped question, “Why study us if you can’t save us?”

PC’s high lethality rate results from its aggressive metastasis coupled with its low probability for diagnosis at an early stage when surgery would have promising curative results. The best prospects for a cure, center on its early detection. Ideally, tests would enable its diagnosis in asymptomatic individuals, because once clinical signs and symptoms of malignancy have manifested, patients may already have a significant tumor burden [3]. At this time, tests for early diagnosis that are cost-effective are not currently available for screening of the general population. However, several authors have described potentially promising findings when focusing cancer control efforts on high-risk populations.

Poley *et al.* [4] studied the use of endoscopic ultrasonography (EUS) for screening of individuals at high risk of developing PC. This included first-degree family members of affected individuals from FPC families and mutation carriers of PC-prone hereditary syndromes such as Peutz-Jeghers syndrome and the FAMMM syndrome. All were asymptomatic and had not undergone EUS in the past. Forty‑four individuals, 18 males and 26 females, aged 32–75 years underwent EUS screening. Results showed “…Three (6.8%) patients had an asymptomatic mass lesion (12, 27, and 50 mm) in the body (n = 2) or tail of the pancreas. All lesions were completely resected. Pathology showed moderately differentiated adenocarcinomas with N1 disease in the two patients with the largest lesions. EUS showed branch-type intraductal papillary mucinous neoplasia (IPMN) in seven individuals.” The authors concluded that screening of individuals at high risk for PC with EUS is not only feasible but also safe. Their findings at first screening were considered to be high where asymptomatic cancer was identified in 7% and premalignant IPMN-like lesions in 16% in their series. Future studies will be required to determine whether such screening improves survival and may help the understanding of the optimal EUS screening interval.

Chu *et al.* [3] reviewed screening studies performed on asymptomatic populations at high risk of PC, one of which was from the University of Washington which involved 14 individuals who were screened with EUS, ERCP, and CT. Interestingly, “…Seven individuals were referred for pancreatectomy based on ERCP abnormalities. These individuals were found to have varying degrees of dysplasia (low-grade to high-grade) on histopathological examination. No individuals had invasive adenocarcinoma or a pathologically normal pancreas.” [5].

A study from Johns Hopkins involved 38 asymptomatic high-risk patients, 37 of whom had familial PC (two or more first- and/or second-degree relatives with PC) and one had Peutz-Jeghers syndrome, each of whom was screened by EUS. Findings showed abnormal EUS exams which were then followed by EUS-FNA, CT, and ERCP. Findings disclosed six individuals with definitive pancreatic lesions (1 invasive ductal adenocarcinoma, 1 IPMN, 2 serous cystadenomas, and 2 non-neoplastic masses). A total of 29 individuals had abnormalities on EUS. Findings disclosed an overall yield of significant masses to be 5.3% (2/38). Noteworthy was a single ductal adenocarcinoma, which was not detected by either the follow-up CT or ERCP evaluations [6].

Our purpose is to update the genetic epidemiology of PC in the interest of advancing progress in its early diagnosis, screening, and management.

## 2. Genetic Epidemiology of Pancreatic Cancer

Review of the epidemiology of PC depicts a disease whose risk correlates with increasing age; only rarely are PC affecteds younger than 25 and it is even relatively uncommon in those younger than 45 years [7]. There is often a male predilection to PC. PC is more common in Western industrialized areas of the world. Geographically and ethnically, some of the world’s highest rates of PC are in New Zealand Maoris, native Hawaiians, and African Americans, with the lowest incidence in inhabitants of India and Nigeria. Jewish people are at a higher risk of PC than other religious groups, while Seventh Day Adventists have an extremely low risk. Its incidence in urban populations is higher than in rural areas. Japanese immigrants to the United States have higher rates of PC than Japanese indigenous to Japan. PC appears to be more common among lower socio-economic classes, indicating a possible link between PC and lifestyle, including food habits, obesity, and other related factors [7].

The genetic epidemiology of PC is exceedingly heterogeneous. For example, Zerón *et al.* [8] discuss such factors as chronic pancreatitis, cigarette smoking, diabetes, obesity, and dietary mutagen exposure in concert with host factors as being some of the most consistent risk factors in the development of PC. Cigarette smoking appears to be the strongest risk factor for PC [9]. Blackford *et al.* [10] note that cigarette smoking doubles the risk of PC, suggesting that smoking accounts for 20% to 25% of PCs. These authors note that PCs in cigarette smokers harbor more mutations than do carcinomas from never-smokers. The types and patterns of these mutations provide insight into the mechanisms by which cigarette smoking causes PC. Heavy alcohol consumption does not appear to pose a significant risk factor, apart from its role in causing pancreatitis which remains under investigation [11].

## 3. Age of PC Onset

Chu *et al.* [3] note that the most significant demographic factor in PC is advancing age and that 80% of all PCs are diagnosed in the age range of 60–80 years [12].

While young age of onset is a hallmark of many hereditary cancer syndromes, the implications of young-onset in familial PC (FPC) family members remains elusive. Brune *et al.* [13] studied age at onset of PC and risk of PC in family members by comparing the observed incidence of PC in 9040 individuals from 1718 kindreds enrolled in the National Familial Pancreas Tumor Registry, to that observed in the general United States population (surveillance, epidemiology, and end results [SEER] registry). Using standardized incidence ratios (SIRs) they found that “…Risk of pancreatic cancer was elevated in both FPC kindred members (SIR = 6.79, 95% confidence interval [CI] = 4.54 to 9.75, P < 0.001) and sporadic pancreatic cancer (SPC) kindred members (SIR = 2.41, 95% CI = 1.04 to 4.74, P = 0.04), compared with the general population. The presence of a young-onset patient (<50 years) in the family, did not alter the risk for SPC kindred members (SIR = 2.41, 95% CI = 0.05 to 15.30, P = 0.59) compared with those without a young-onset case in the kindred (SIR = 2.36, 95% CI = 0.95 to 4.88, P = 0.06). However, risk was higher among members of FPC kindreds with a young-onset case in the kindred (SIR = 9.31, 95% CI = 3.42 to 20.28, P < 0.001) than those without a young-onset case in the kindred (SIR = 6.34, 95% CI = 4.02 to 9.51, P < 0.001).” It was of interest that the youngest age of PC in the kindred did not affect the risk among SPC kindred members. These authors conclude that individuals with a family history of PC are at a statistically increased risk of developing PC but having a member of the family with a young-onset PC confers an added risk in FPC kindreds.

## 4. Family History

There has been a recent groundswell of knowledge about hereditary forms of cancer, although its translation into the clinical setting has been problematic. For example, a comprehensive history of cancer in a family, often the linchpin to this effort, has been insufficiently recorded in many patients’ medical records, thereby compromising its clinical significance [14]. Obstacles are not just a problem for physicians; they also involve families who must participate actively in the process of becoming aware of their family cancer histories and, once diagnosed with a cancer causing hereditary syndrome, they must comply with surveillance and management recommendations, if cancer control is to achieve success.

Shi *et al.* [15] emphasize the extreme importance of collecting a comprehensive cancer family history, including cancer of all anatomic sites, when considering that approximately 5% to 10% of individuals with PC report a history of this disease in a close family member. Most PCs arising in patients with a family history are ductal adenocarcinomas. However, certain subtypes of PC may be associated with hereditary PC-prone syndromes, thereby mandating careful pathologic review of each case since the histologic appearance of the PC may prove valuable for elucidating its clinical index of suspicion for its hereditary basis.

## 5. Genetic Counseling

Many high-risk patients can benefit immensely from genetic counseling. This becomes of extreme importance when a cancer-causing germline mutation has been identified in a family, such as the *CDKN2A* mutation in the familial atypical multiple mole melanoma-pancreatic cancer (FAMMM-PC) prone syndrome [2,16,17,18]. Specifically, patients need to know about the natural history of the hereditary disorder afflicting their family, the logic involved in screening and management and, importantly, the psychological burden associated with their potential cancer lifetime destiny. They may also experience guilt should they be found to have inherited the wild-type gene: “Why was I spared this tragic event when my siblings and other family members received the bad news?”

## 6. Pathology of PC

Real *et al.* [19] stressed the importance of gaining a better understanding of the genetic and environmental carcinogenic factors which predispose to PC, in concert with those pathogenetic mechanisms involved in its development and progression. They considered three major advances which have taken place during the past decade and which may be paving the road to its understanding. These involve “…(1) the establishment of consensus pathologic definitions for pancreatic intraepithelial neoplastic (PanIN) lesions; (2) the identification of the major genes involved in pancreatic cancer, and (3) the development of novel mouse models of disease that mimic human PDAC [pancreatic ductal adenocarcinoma].”

Hruban *et al.* [20] postulate that PC arises from morphologically distinct non-invasive precursor lesions which include “…the intraductal papillary mucinous neoplasm, the mucinous cystic neoplasm, and pancreatic intraepithelial neoplasia.”

Hruban *et al.* [21] note that pancreatic intraepithelial neoplasia (PanIN) is a well-defined histological precursor to invasive ductal adenocarcinoma and is a remarkably common lesion in elderly individuals. The progression of PanIN to invasive cancer has been aided by molecular studies. Genetically engineered mouse models have recently been generated that “…recapitulate the entire spectrum of lesions from precursor to invasive pancreatic cancer. Some PanIN lesions produce lobulocentric atrophy of the pancreatic parenchyma, and, when multifocal, this lobulocentric atrophy may be detectable using currently available imaging techniques such as endoscopic ultrasound. The association of acinar-ductal metaplasia with PanIN lesions has led some to hypothesize that PanINs develop from acinar cells that undergo acinar-ductal metaplasia [21].

Hruban *et al.* [22] also note that PC is fundamentally caused by mutations in specific genes which have been studied during the past decade. Their position is that better comprehension of these genes, as well as their function, should not only identify familial forms of PC but moreover should “…define the precursor lesions from which invasive pancreatic cancers arise, and will soon lead to gene-specific therapies for this disease...”.

Sipos *et al.* [23] note that while most PCs are classified as ductal adenocarcinomas, it is important to note that ductal lesions may give rise to a pancreatic ductal adenocarcinoma referred to as the mentioned pancreatic intraepithelial neoplasia (PanIN). This classification system distinguishes among three grades of PanIN. Molecular studies have shown that PanIN-2 and PanIN-3 lesions represent a distinct step on the way to invasive PC. Such PanIN of high grade lesions are extremely rare in the normal pancreas. However, low-grade PanINs are common in patients older than age 40. In addition, they “…may be associated with lobular fibrosis and intraductal papillary mucinous neoplasms of the gastric type. This disease spectrum has also been described in members of kindreds with familial pancreatic cancer…” The authors conclude that it would be helpful to target PanIN-2 lesions in the context of being the starting point of progressive neoplastic changes leading to invasive pancreatic ductal adenocarcinoma. They also suggest that PanINs may be used as potential biomarkers to facilitate diagnosis and therapy.

Sparr *et al.* [24] discuss IPMN of the pancreas as a precancerous lesion which shows progression to carcinoma. They report a 61-year-old woman with a phenotype consistent with LS and a confirmed *MSH2* germline mutation. Interestingly, the patient’s adenocarcinoma of the colon and IPMN of the pancreas revealed identical immunohistochemical staining profiles showing loss of expression of MSH2 and MSH6 proteins with high levels of MSI. The authors concluded that “…The immunohistochemical staining and microsatellite instability patterns of the adenocarcinoma of the colon and IPMN provides strong evidence to support the consideration of IPMN as part of the spectrum of lesions found in LS.

## 7. Genetics

Numerous case-control studies have described families with two or more first-degree relatives with PC which fit a familial category [7]. When such families are extended, several studies [16,25,26,27] have shown a pattern of PC in a subset suggestive of an autosomal dominantly inherited factor. As opposed to a familial risk category of PC, approximately 5–10% of PCs have a hereditary basis and may interact strongly with endogenous and exogenous risk factors.

Landi [28] has described an extensive array of hereditary cancer-prone syndromes involving PC as an integral lesion (see Table 1). Not unexpectedly, most of the deleterious genes responsible for various well-defined cancer syndromes such as *CDKN2A* for the FAMMM syndrome, mismatch repair (MMR) for Lynch syndrome, *TP53* for Li-Fraumeni syndrome, *APC* for familial adenomatous polyposis, and *BRCA2* for the hereditary breast-ovarian cancer syndrome, indicate that the PC is part of each disorder’s cancer spectrum. Landi [28] has also ranked known/possible risk factors through extending analysis to hereditary pancreatitis, diabetes, or specific environmental exposures such as smoking. Furthermore, Landi [28] notes that recent work has revealed new genes that are somatically mutated in PC, including alterations within the pathways of Wnt/Notch and DNA MMR. Kastrinos *et al*. [29] provide estimates for a cumulative risk of PC of 1% by age 50 and 3.68% by age 70.

Jones *et al.* [30] performed a comprehensive genetic analysis of 24 advanced PCs wherein they determined the sequences of 23,219 transcripts representing 20,661 protein-coding genes in the samples. Homozygous deletions and amplifications in the DNA of these tumors were investigated through the use of microarrays containing probes for approximately 10^6^ SNPs. Findings disclosed that “…pancreatic cancers contain an average of 63 genetic alterations, the majority of which are point mutations. These alterations defined a core set of 12 cellular signaling pathways and processes that were each genetically altered in 67 to 100% of the tumors. Analysis of these tumors’ transcriptomes with next-generation sequencing-by-synthesis technologies provided independent evidence of the importance of these pathways and processes…” These genetically altered core pathways and regulatory processes only became evident when the coding regions of the genome were analyzed in depth. Furthermore, “…Dysregulation of these core pathways and processes through mutation can explain the major features of pancreatic tumorigenesis.” Jones *et al.* [30] indicate that genetic alterations in the *CDKN2A*, *SMAD4*, and *TP53* tumor suppressor genes and in the *KRAS* oncogene have been identified in this lethal cancer. They emphasize that these discoveries, important in comprehending the natural history of PC, spurred efforts for developing improved diagnostic and therapeutic opportunities, since the vast majority of human genes have not been analyzed in this particular cancer. Recognizing that all human cancers are primarily genetic disorders, their plan is to identify additional genes and signaling pathways in the hope that this effort will guide future research on PC. They concluded that the key to understanding the pathogenesis of PCs rests on an appreciation of a core set of genetic pathways and processes. Importantly, they identified 12 partially overlapping processes that are genetically altered in the great majority of PCs; nevertheless, the pathway components that are altered in each tumor vary widely. For example, two of the tumors each contained mutations of a gene involved in the TGF-β pathway, one being *SMAD4* and the other being *BMPR2*. Interestingly, these two tumors each contained mutations of genes involved in most of the other 11 core processes and pathways, but the genes altered in each tumor were largely different. However, from the practical standpoint Jones *et al.* [30] indicate that, while their data yielded insights into tumor pathogenesis, their research also provided data required for personalized cancer medicine. For example, when compared to certain forms of leukemia, wherein tumorigenesis may be driven by a single targetable oncogene, these authors reasoned that PCs result from genetic alterations in a large number of genes and that they function through a relatively small number of pathways. They also suggest that “…the best hope for therapeutic development may lie in the discovery of agents that target the physiologic effects of the altered pathways and processes rather than their individual gene components. Thus, rather than seeking agents that target specific mutated genes, agents that broadly target downstream mediators or key nodal points may be preferable…”

**Table 1:** Risk factors for PC according to known diseases, familial factors, or environmental exposures. Classes of risk are categorized, arbitrarily, as reported in Section 5. Abbreviations used: Standardized Incidence Rate (SIR), Odd Ratio (OR), Relative Risk (RR), Confidence Interval (CI). (Reprinted from [28], Copyright 2009, with permission from Elsevier.)

Risk condition	Risk measure	Class of risk	Reference
**Familial atypical mole-multiple melanoma (FAMMM)/ Melanoma-pancreatic cancer syndrome**			
*CDKN2A*	17% of patients (95% CI 13–30) develop PC by the age of 75 (*vs* 0.53–0.85% in non carriers). RR = 20	Very high	PMID:10956390
*CDKN2A*	SIR = 38 (95% CI, 10–97), in patients also with melanoma SIR = 52 (95% CI, 14–133)	Very high	PMID:15173226
*CDKN2A*	13.4-fold increase	Very high	PMID: 2372499
**Peutz-Jeghers syndrome**			
*STK* *11*	4% of patients develop PC by 40 years and 8% by 60 years	Very high	PMID: 15188174
**Hereditary pancreatitis (HP)**			
Any of *PRSS1, SPINK1, PRSS2, CTRC*	The risk ranges between 26-fold to 60-fold, with a cumulative risk of 40% by age 70	Very high	PMID: 18184119 PMID: 10872429 PMID: 10872414
**Cystic Fibrosis**			
*CFTR*, patients affected	OR = 31.5 (95% CI 4.8–205)	Very high	PMID: 7830730 PMID: 8217592
**Familial pancreatic cancer (not attributable to the other ascertained cancer syndromes).**			
*For three or more first-degree relatives*	SIR = 32 (95% CI, 10.4–74.7)	Very high	PMID: 15059921
***PALLD*-dependent familial PC**			
*PALLD*	Rare. Found in 1 of 84 probands with familial PC. It explains an irrelevant fraction of familial PCs.	Very high	PMID:11474289 PMID:17415588
**Endocrine pancreas: Multiple Endocrine Neoplasia**			
*Any of MEN1, RET, CDKN1B*	-	Limited data suggestive of very high risk	PMID: 7913018
**Endocrine pancreas: ** **Von Hippel-Lindau syndrome (VHL)**			
*VHL*	17% of VHL probands show PC	Limited data suggestive of very high risk	PMID:573913
**Lynch syndrome (human non-polyposis colorectal cancer; HNPCC)**			
*MLH1*	Familial RR = 5.6(p < 0.05) OR = 7.6(NS)	High	PMID:17939062
*MSH2*	Familial RR = 2.3 (NS) OR = 7.9(NS)	Limited data suggestive of high risk	PMID: 17939062
*MSH6*	inconclusive	Limited data, likely high risk	PMID:17939062
**Breast/Ovarian familial cancer**			
*BRCA2*	RR ranging from 5.9 to 10	High	PMID:17148771 PMID:16141007 PMID:15516847
**Familial pancreatic cancer (not attributable to the other ascertained cancer syndromes).**			
*For two first-degree relatives*	SIR = 6.4 (95% CI, 1.8–16.4)	High	PMID: 15059921
*when smoking+ one first-degree relative with PC*	RR = 6.02 (95% CI 1.98–18.29)	High	PMID: 12670518
**Li Fraumeni syndrome**			
*TP53*	1.3% of all cancers in Li Fraumeni patients are PC	Limited data suggestive of high risk	PMID: 9006316
**Familial adenomatous polyposis (FAP)**			
*APC*	RR ranging from 4.46 (95% CI, 1.2–11.4) to RR = 5	Intermediate	PMID:15516847 PMID: 8244108
**Familial pancreatic cancer (not attributable to the other ascertained cancer syndromes).**			
*For one first-degree relative*	SIR = 4.5 (95% CI, 0.54–16.3)	Intermediate	PMID: 15059921
**Non hereditary pancreatitis**			
	4% cumulative lifetime risk of PC in patients with any form of pancreatitis	Intermediate	PMID: 8479461
*>7 years of pancreatitis*	RR = 2.04 (95% CI: 1.53–2.72)	Low	PMID: 7797022
**Breast/Ovarian familial cancer**			
*BRCA1*	RR = 3.1 (95% CI 0.45–21)	Limited data suggestive of low risk	PMID:17148771
**Cystic Fibrosis**			
*CFTR*, carriers + young onset PC (<60 years)	OR = 2.18 (1.24–3.29)	Low	PMID: 16227367
**Diabetes**			
	RR ranges between 2.1 (95% CI 1.6–2.8) and 2.6 (95%CI 1.6–4.1)	Low	PMID: 7745774 PMID: 1287744
	SIR = 2.1 (95% CI 1.9–2.4)	Low	Very large prospective cohort study (20,475 men and 15,183 women) in the US.
**Smoking habit**			
	RR ranges between 2.5 (95% CI 1.9–3.2) to 2.70 (95% CI: 1.95–3.74)	Low	PMID: 12670518
**Diet and nutrition**			
*Excessive dietary consumption of: dairy products, eggs, milk, fried food, low fresh fruit and vegetable consumption, low fibers, salted foods, smoked meat dehydrated foods and fried foods*	RR in the range between 3.10 (95% CI 1.55–6.22) for dehydrated foods, up to 4.68 (95% CI 2.05–10.69) for smoked meat	Low/Intermediate	PMID: 12670518
**Diskeratosis congenital**			
*DKC1*	Case report: occurrence of one case of PC in one large pedigree	Limited data, unknown	PMID: 7272212
**Palmoplantar keratoderma**			
*Type I acidic keratin gene cluster*	Case report: occurrence of PC in one large pedigree	Limited data, unknown	PMID: 8733379
**Alcohol and coffee drinking**			
*Excluding people with chronic alcoholic pancreatitis*	inconclusive	Likely no risk	PMID: 12670518
**Ataxia Telangiectasia (AT)**			
*ATM, carriers*	2.41 (95% CI 0.34–17.1)	Limited data suggestive of no risk	PMID: 15928302

## 8. Extra-Pancreatic Cancers in Hereditary PC

Wang *et al.* [31], when considering that hereditary cancer syndromes are often characterized by familial clustering of variable organ sites, investigated whether cancers other than PC cluster in PC‑prone kindreds. Utilizing mortality patterns among the relatives of National Familial Pancreatic Tumor Registry probands, which included over 200,000 person-years of follow-up from 8,564 first-degree relatives of probands and 1,007 spouse controls as part of their analysis, they found that cancer (all types) mortality was increased in the relatives of sporadic probands (weighed standardized mortality ratio [wSMR]1.55, 95% CI 1.39–1.73) and familial probands (wSMR 1.41, 95% CI 1.26–1.58). For example, “…Relatives of familial probands had a significantly increased risk of dying from breast (wSMR 1.66, 95% CI1.15–2.34), ovarian (wSMR 2.05, 95% CI 1.10–3.49), and bile duct cancers (wSMR 2.89, 95% CI 1.04–6.39). Relatives of sporadic probands were at increased risk of dying from bile duct cancer (wSMR 3.01, 95% CI 1.09–6.67). Relatives of young onset probands were at higher risk of dying from prostate (wSMR 2.31, 95% CI 1.14–4.20). Increased cancer mortality was not observed in the spouse controls. [Their] results show that relatives of pancreatic cancer patients are at higher risk of developing cancers at other sites and highlight the importance of complete family history in clinical risk assessment.” Carcinoma of the breast and ovary, in variable association with PC, is now well established in patients/families with *BRCA* germline mutations. Al-Sukhni *et al.* [32] note that the association of germline *BRCA2* germline mutations with PC has been well established. However, the role of its *BRCA1* counterpart mutations is less clear. Their study disclosed that the loss of heterozygosity at the BRCA1 locus “…occurs in pancreatic cancers of germline *BRCA1* mutation carriers, acting as a ‘second-hit’ event contributing to pancreatic tumorigenesis.” They concluded that *BRCA1* germline mutations may be considered for PC screening. Similar results were found among *BRCA1*-prone families by Lynch *et al.* [33].

Couch *et al.* [34] have recently identified breast cancer susceptibility loci through genome-wide association studies and they evaluated possible associations between these single-nucleotide polymorphisms (SNPs) and PC risk. They concluded that “Association studies in a large pancreatic case-control study indicate that SNPs associated with breast cancer may also be associated with pancreatic cancer susceptibility and survival.”

Hiripi *et al.* [35] discuss the familial association of PC with other malignancies using the updated Swedish Family-Cancer Database which includes more than 11.5 million individuals. Findings disclosed that the risk of PC was elevated in those patients “…with a parental history of cancers of the liver (RR 1.41; 95% CI 1.10–1.81), kidney (RR 1.37; 95% CI 1.06–1.76), lung (RR 1.50; 95% CI 1.27–1.79), and larynx (RR 1.98; 95% CI 1.19–3.28). ...parental history of pancreatic cancer and cancers of the small intestine, colon, breast, lung, testis and cervix in offspring. There was an increased risk of pancreatic cancer associated with early-onset breast cancer in siblings.” The authors appropriately conclude that smoking may contribute to the familial aggregation of PC and lung tumors, while familial clustering of PC and breast cancer could be partially explained by mutations in the *BRCA2* gene.

## 9. Familial Atypical Multiple Mole Melanoma (FAMMM) Syndrome

Lynch *et al.* were the first to describe pancreatic cancer as an integral lesion in the FAMMM syndrome [17,18,36,37]. The FAMMM-pancreatic carcinoma (FAMMM-PC) association in concert with the *CDKN2A* germline mutation was described within these families [38]; malignant melanoma predominated in certain of them whereas PC predominated in others. Early-onset PC, as early as age 35 years, appeared in some of the families in contrast to markedly later-onset PC in others.

**Figure 1:**
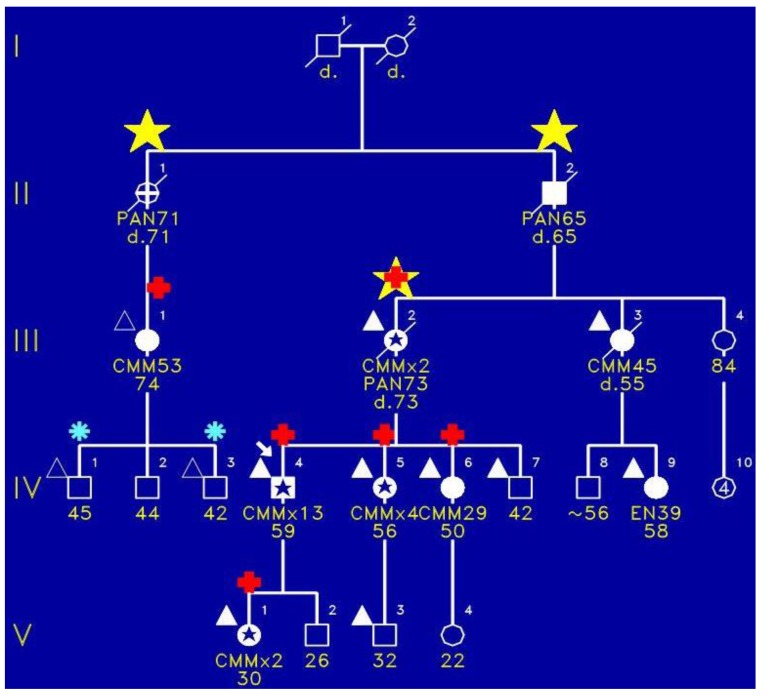
Pedigree of FAMMM syndrome with yellow star indicating pancreatic cancer, red + sign showing presence of *CDKN2A* pathogenic mutation, blue asterisk indicating tested but no pathogenic mutation, and triangles showing typical FAMMM cutaneous features.

**Figure 2:**
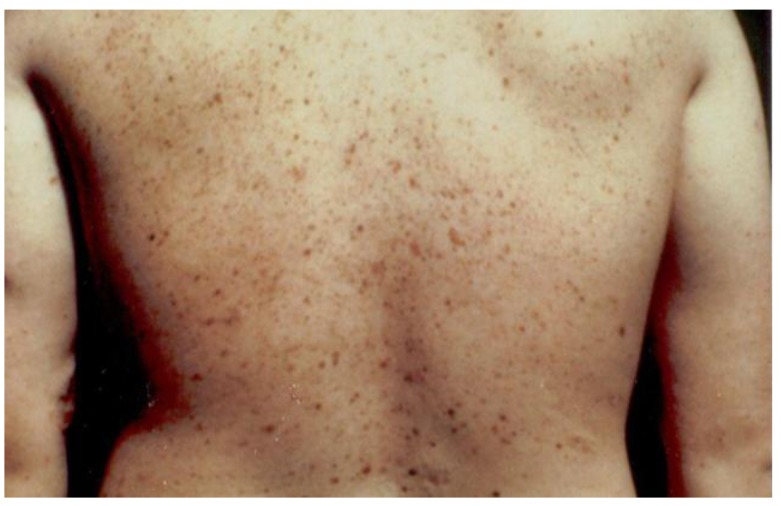
Photograph of the proband with multiple atypical nevi characterized by variegated coloration, irregular contours, and larger size than typical moles.

Figure 1 portrays the initial family in which we identified the FAMMM syndrome. This family has three cases (yellow stars) of pancreatic cancer wherein III-2 had both the FAMMM phenotype and pancreatic cancer, as did her father (II-2) and a paternal aunt (II-1). The red + signs indicate the presence of *CDKN2A* germline mutation which is believed to be the pathogenic causal mutation for the syndrome in this family. CMM indicates melanomas and it is noteworthy that the proband had 13 melanomas. Figure 2 is a photograph of the proband (Figure 1, IV-1) showing multiple atypical nevi which are irregular-appearing moles with variegation in coloration, often sizable, and with irregular contours.

**Figure 3:**
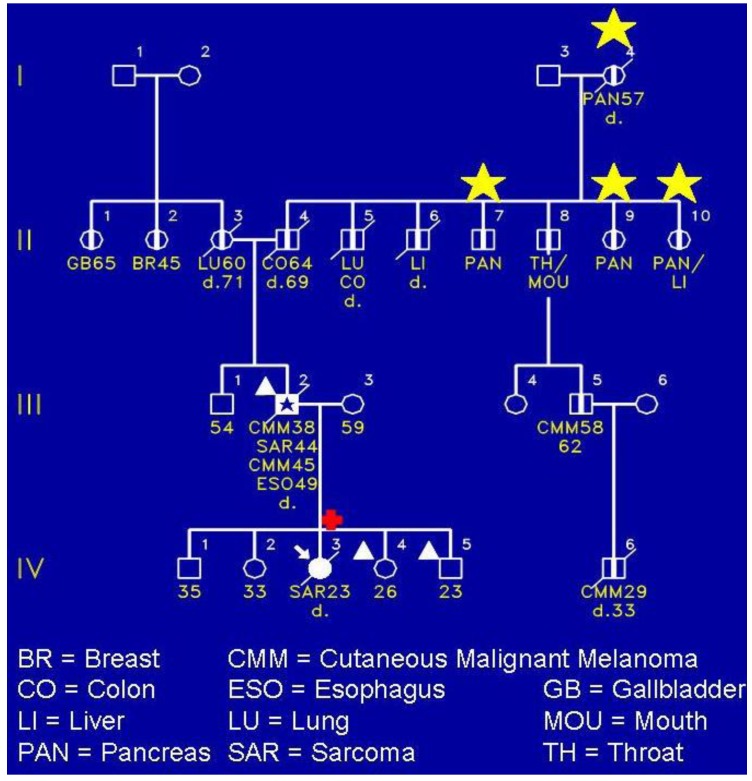
A FAMMM syndrome affected (III-2) patient with cutaneous malignant melanoma, sarcoma, and esophageal carcinoma, and daughter (IV-3) with *CDKN2A* pathogenic mutation but no evidence of FAMMM phenotype. The daughter had the *CDKN2A* mutation and died of a sarcoma of the right shoulder at age 23. Her sister (VI-4) and brother (IV-5) each had FAMMM phenotype but have not yet been tested for the mutation.

Figure 3 shows a FAMMM family wherein the proband (IV-3) died of a sarcoma of her shoulder. She had the *CDKN2A* germline mutation. Of keen interest is that she did not have any evidence of the FAMMM phenotype comprised of multiple atypical nevi. However, her father (III-2) had the FAMMM phenotype, two melanomas, a sarcoma, and he died of esophageal cancer. He was a nonsmoker and not a consumer of alcohol. It is noteworthy that the proband’s paternal lineage showed four cases of pancreatic cancer as evidenced in II-7, II-9, II-10, and the proband’s paternal great‑grandfather (I-4). The yellow stars indicate the presence of pancreatic cancer.

**Figure 4:**
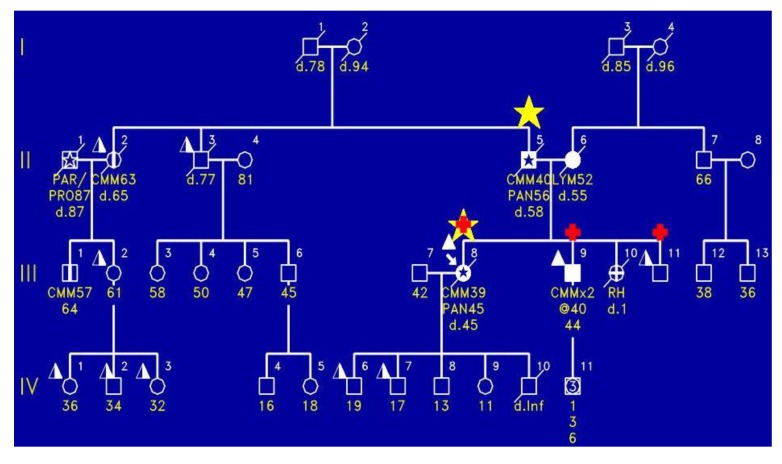
Shows the proband (III-8) to have the FAMMM phenotype, manifesting malignant melanoma at age 39 and dying of pancreatic cancer at age 45; he had the *CDKN2A* mutation. There are two brothers (IV-9 and IV-10) who had the FAMMM phenotype and each had the *CDKN2A* mutation. The father of this sibship had pancreatic cancer and cutaneous malignant melanoma. Photograph of the proband with multiple atypical nevi characterized by variegated coloration, irregular contours, and larger size than typical moles.

Figure 4 shows the proband (III-8) with the FAMMM syndrome with malignant melanoma, who died of pancreatic cancer at age 45. As is indicated by the red + signs, the proband had the *CDKN2A* mutation as did two of the siblings (III-9, III-11). Importantly, the proband’s mother had melanoma and died of pancreatic cancer.

## 10. PC in Lynch Syndrome

Gargiulo *et al.* [39] note that only a small subset of PC cases harbor pathogenic MMR mutations.

Kastrinos *et al.* [29] studied 147 families with germline MMR mutations and compared the risk of PC in these families with the risk of PC in the general United States population. Their findings showed that PC in these families exceeded expectations from the general population and therefore “…Individuals with MMR gene mutations and a family history of pancreatic cancer are appropriate to include in studies to further define the risk for premalignant and malignant pancreatic neoplasms and potential benefits and limitations of surveillance.”

## 11. *PALLD* Gene

The issue of the role of *PALLD*, the gene that encodes the protein palladin, in PC is controversial. More research is clearly needed. The heterogeneity of hereditary and familial forms of PC must be considered, given the possibility that the palladin protein may be involved in some forms of PC but not in others.

The palladin gene (*PALLD*) was identified by Pogue-Geile *et al.* [40] who described a germline missense alteration (P239S) in that gene in a familial PC family. They suggested that this variant may form the basis of familial clustering of PC for early-onset PC with pancreatic insufficiency and diabetes mellitus. The family appeared to behave as an autosomal dominant with significant linkage to chromosome 4q32-34, a region where the *PALLD* gene is located. This subject is reviewed by Klein *et al.* [41] who indicate that Pogue-Geile *et al.* “…implicated an oncogenic function for palladin after finding overexpression of *PALLD* mRNA in pancreatic cancer tissues.” However, Klein *et al.* note that since the Pogue-Geile *et al.* paper was published, subsequent investigations failed to find evidence linking palladin to familial PC. Furthermore, Klein *et al.* note that investigators have failed to evaluate the full sequence of *PALLD* in patients with familial PC in order to determine if sequence variants in *PALLD* might be contributing to PC susceptibility. Given this background, Klein *et al.* sequenced the entire coding region of *PALLD* in 48 individuals with familial PC. Importantly, they did not find any deleterious mutations and were not able to show evidence to implicate mutations in *PALLD* as a cause of familial PC.

Salaria *et al.* [42] conclude that “The overexpression of palladin relative to normal pancreas in the majority of pancreatic cancers is limited to non-neoplastic stromal cells.” These authors conclude that, given the fact that palladin is not overexpressed in most PC cells, it then follows that palladin’s overexpression is not likely to impact the invasive and migratory abilities of PC cells.

In contrast, a more recent study identified the actin-associated protein palladin as a potential candidate biomarker in pancreatic ductal adenocarcinoma (PDA); the protein was found to be overexpressed in a rare inherited form of PDA. It is also overexpressed in a number of sporadic pancreas tumors as well as in premalignant precursors [43]. For example, they note that “…The 85–90 kDa palladin isoform is highly overexpressed in tumor-associated fibroblasts (TAFs) in both primary and metastatic tumors compared to normal pancreas, in samples obtained from either human patients or genetically engineered mice. …upregulation of 85–90 kDa palladin isoform may play a role in the establishment of the TAF phenotype, and thus in the formation of a desmoplastic tumor microenvironment.” The authors conclude that palladin may play a potential role in the early diagnosis of PDA. They suggest that the precise role of palladin in PC has yet to be defined while, as mentioned, *PALLD* is associated with a rare form of familial PC where palladin was identified as being overexpressed in samples of sporadic PC as well as in tumor-derived cell lines [40], results which were challenged in a subsequent investigation that utilized immunohistochemical (IHC) screenings of the pancreas tumor array [42]. Goicoechea *et al.* [43], commenting on this matter, note that these results “…provide evidence that palladin is overexpressed specifically in pancreas tumors, yet the identity of the cell type that is responsible for upregulating palladin in these tumors remains unclear.” Goicoechea *et al.* [43] note that there are three major palladin isoforms which arise from alternative start sites and multiple minor isoforms resulting from alternative splicing giving rise to a rich diversity of isoforms and therein “…raises the possibility that human cells may express palladin variants that are not detected by all antibodies, which could be the cause of previous conflicting results…” These authors then show two major isoforms of palladin in pancreatic tumors, namely 65 kDa and 85–90 kDa. Therein, “…PDA expresses predominantly the 85–90 kDa isoform of palladin, while normal pancreas and non-PDA tumors both express the 65 kDa isoform…” Furthermore, they show “…that palladin overexpression occurs primarily in tumor-associated fibroblasts (TAFs), and not the neoplastic epithelial cells, of human pancreatic tumors. These results suggest the possibility that upregulation of 85–90 kDa palladin may be a critical step in the acquisition of the activated fibroblast phenotype, which is key to the formation of a pro-invasive tumor microenvironment.” Clearly, palladin’s role, if any, in familial/hereditary PC remains an intriguing research question.

## 12. *PALB2* Gene

Recently, Jones *et al.* [44] identified mutations in *PALB2* which may be a PC susceptibility causative germline mutation. A study by Slater *et al.* [45] suggests that *PALB2* mutations might be causative for familial PC in a small subset of European families, especially in those also manifesting breast cancer. More research is clearly needed.

## 13. Aberrant DNA Methylation and MicroRNA Expression

Li *et al.* [46] discuss the importance of aberrant DNA methylation coupled with microRNA expression in the pathogenesis of PC. Their findings showed that most PCs express *miR-200a* and *miR-200b*, but this expression did not affect *SIP1* expression, since “…the *SIP1* promoter is silenced by hypermethylation and in these cancers *E-cadherin* is generally expressed. Both *miR-200a* and *miR200b* were significantly elevated in the sera of pancreatic cancer and chronic pancreatitis patients compared with healthy controls (*P* ≤ 0.0001), yielding receiver operating characteristic curve areas of 0.861 and 0.85, respectively…” These authors concluded that most PCs display hypomethylation in concert with overexpression of *miR-200a* and *miR-200b* silencing of *SIP1* promoter methylation and retention of *E-cadherin* expression. Of major clinical importance is their suggestion that elevated serum levels of *miR-200a* and *miR-200b* in the majority of patients with PC might harbor diagnostic potential.

## 14. PancPRO

PancPRO is the first risk prediction model for PC which is based upon findings from one of the largest registries of familial PC enabling accurate risk assessment [47]. It has been described as a Mendelian risk prediction tool for PC, which was built on the BRCAPRO model [48]. The authors note that “…Using family history of pancreatic cancer, PancPRO estimates the probability that an individual carries a pancreatic cancer susceptibility gene and the future probability that an asymptomatic individual will develop pancreatic cancer. [They] validated the PancPRO model using data on 961 families enrolled in the National Familial Pancreatic Tumor Registry.”

## 15. Markers for Diagnosis of PC

There are few biomarkers which enable high sensitivity and specificity for early diagnosis of PC. Kim *et al.* [49] have shown poor positive predictive value of CA 19-9 for the diagnosis of PC in asymptomatic patients. Märten *et al.* [50] investigated soluble iC3b as an early marker for PC and found it to be superior to both CA19.9 and radiology. Sensitivity and specificity of iC3b could be increased by combining it with CA19.9. Screening for siC3b in patients at an increased risk for pancreatic ductal carcinoma allowed for its early detection with high sensitivity.

## 16. Future Directions

Creating hypotheses for reducing PC morbidity and mortality is clearly an exceedingly difficult task. The most promising areas seem to be in improved imaging or in new gene probes.

Decker *et al.* [51] have recently assessed imaging modalities for screening of PC. Computed tomography has poor sensitivity and there is concern about radiation exposure with repeated use in high-risk groups. Positron emission tomography lacks anatomic detail (though receptor-targeted imaging of PC has shown promise in a mouse model [52]). The role of magnetic resonance imaging continues to evolve with technological advancements, ERCP has significant morbidity.

The most sensitive imaging modality for the diagnosis of PC is probably endoscopic ultrasound (EUS), though there is poor interobserver agreement on EUS interpretations [53]. Kimmey *et al.* [54] reported in 2002 a series of 46 patients undergoing EUS: 28 patients had ERCPs, leading to 12 pancreatectomies (all with widespread dysplasia, none with adenocarcinoma). Previously, Canto *et al.* [6] showed that EUS screening of asymptomatic high-risk individuals can detect resectable neoplasm, and more recently [55] described 78 high-risk patients in which screening found eight patients with neoplasia. EUS abnormalities suggestive of chronic pancreatitis were identified more commonly in high-risk individuals. Kluijt *et al.* [56] have described apparently successful EUS screening in three FAMMM family members.

Others have tried to find diagnostic clues through analysis of pancreatic juice. Yan *et al.* [57] looked at chromosomal DNA extracted from pancreatic juice. Absence of certain abnormalities suggested negligible risk of PC. Nakashima *et al.* [58] analyzed pancreatic juice for human telomerase reverse transcriptase (hTERT). It was detected preoperatively in 84% of adenocarcinoma cases. Recently, Zhang *et al.* [59] found salivary messenger-RNA biomarkers (KRAS, MBD3L2, ACRV1, and DPM1) could differentiate PC patients from those with chronic pancreatitis and from healthy controls.

## 17. Conclusion

In conclusion, Chu *et al.* [3] note that the five year survival rate in PC is only 5%, which is the lowest of all malignancies, given the fact that the majority of PC affected individuals already have metastatic disease at the time of diagnosis and only 15%–20% are determined to be surgically resectable [60]. The median survival of PC has been shown to be 2.5–8 months without surgical resection which improves to 13–21 months with surgical resection [60]. Clearly, surgical resection has been the strongest hope as lifesaving therapy for PC, since at the present time there is no effective screening test for PC.

Chu *et al.* [3] call attention to the encouraging fact that with recognition of early stage cancers the five year survival rate improved to 32%, while lymph node-negative, margin-negative, PCs improved to 41% [61]. In addition, neoadjuvant gemcitabine-based chemoradiation in stage I and II cancers showed a median survival time of 34 months with a five year survival rate of 36% in patients who underwent pancreaticoduodenectomy [62,63]. More research will be required in order to determine survival benefit through screening of high-risk patients.

Why are we addressing statistics on the one hand that are discouraging, as in the case of PC’s late diagnosis with metastatic spread, *versus* relative encouragement when detected early, particularly in a stage I disease? One answer, from our perspective, rests upon the need to focus heavily upon individuals at inordinately high risk for PC when based upon environmental and genetic factors and their interaction. Such patients will then be the ones who will be candidates for innovative approaches to safe screening measures and, in turn, they will be high order candidates for molecular genetics, pathophysiology, pathology, and environmental modification, and genetic investigations.

## References

[1] Jemal A., Siegel R., Ward E., Hao Y., Xu J., Thun J.T. (2009). Cancer Statistics, 2009. CA Cancer J. Clin..

[2] Lynch H. T., Brand R.E., Lynch J.F., Fusaro R.M., Smyrk T.C., Goggins M., Kern S.E. (2000). Genetic counseling and testing for germ-line *p16* mutations in two pancreatic cancer-prone families. Gastroenterology.

[3] Chu D., Kohlmann W., Adler D.G. (2010). Identification and screening of individuals at increased risk for pancreatic cancer with emphasis on known environmental and genetic factors and hereditary syndromes. JOP.

[4] Poley J.W., Kluijt I., Gouma D.J., Harinck F., Wagner A., Aalfs C., van Eijck C.H.J., Cats A., Kuipers E.J., Nio Y., Fockens P., Bruno M.J. (2009). The yield of first-time endoscopic ultrasonography in screening individuals at high risk of developing pancreatic cancer. Am. J. Gastroenterol..

[5] Brentnall T.A., Bronner M.P., Byrd D.R., Haggitt R.C., Kimmey M.B. (1999). Early diagnosis and treatment of pancreatic dysplasia in patients with a family history of pancreatic cancer. Ann. Intern. Med..

[6] Canto M.I., Goggins M., Yeo C.J., Griffin C., Axilbund J.E., Brune K., Ali S.Z., Jagannath S., Petersen G.M., Fishman E.K., Piantadosi S., Giardiello F.M., Hruban R.H. (2004). Screening for pancreatic neoplasia in high-risk individuals: An EUS-based approach. Clin. Gastroenterol. Hepatol..

[7] Lynch H.T., Smyrk T., Kern S.E., Hruban R.H., Lightdale C.J., Lemon S.J., Lynch J.F., Fusaro L.R., Fusaro R.M., Ghadirian P. (1996). Familial pancreatic cancer: a review. Sem. Oncol..

[8] Zerón H.M., GarcÍa Flores J.R., Romero Prieto M.L. (2009). Limitations in improving detection of pancreatic adenocarcinoma. Future Oncol..

[9] Lowenfels A.B., Maisonneuve P., Whitcomb D.C., Lerch M.M., DiMagno E.P. (2001). Cigarette smoking as a risk factor for pancreatic cancer in patients with hereditary pancreatitis. JAMA.

[10] Blackford A., Parmigiani G., Kensler T.W., Wolfgang C., Jones S., Zhang X., Parsons D.W., Lin J.C.-H., Leary R.J., Eshleman J.R., Goggins M., Jaffee E.M., Iacobuzio-Donahue C.A., Maitra A., Klein A., Cameron J.L., Olino K., Schulick R., Winter J., Vogelstein B., Velculescu V.E., Kinzler K.W., Hruban R.H. (2009). Genetic mutations associated with cigarette smoking in pancreatic cancer. Cancer Res..

[11] Lowenfels A.B., Maisonneuve P., DiMagno E.P., Elitsur Y., Gates L.K., Perrault J., Whitcomb D.C., International Hereditary Pancreatitis Study Group (1997). Hereditary pancreatitis and the risk of pancreatic cancer. J. Natl. Cancer Inst..

[12] Lillemoe K. D., Yeo C.J., Cameron J.L. (2000). Pancreatic cancer: state-of-the-art care. CA Cancer J. Clin...

[13] Brune K.A., Lau B., Palmisano E., Canto M., Goggins M.G., Hruban R.H., Klein A.P. (2010). Importance of age of onset in pancreatic cancer kindreds. J. Natl. Cancer Inst..

[14] Guttmacher A.E., Collins F.S., Carmona R.H. (2004). The family history -- more important than ever. N. Engl. J. Med..

[15] Shi C., Hruban R.H., Klein A.P. (2019). Familial pancreatic cancer. Arch. Pathol. Lab. Med..

[16] Lynch H.T., Fusaro R.M. (1991). Pancreatic cancer and the familial atypical multiple mole melanoma (FAMMM) syndrome. Pancreas.

[17] Lynch H.T., Frichot B.C., Lynch P., Lynch J., Guirgis H.A. (1975). Family studies of malignant melanoma and associated cancer. Surg. Gynecol. Obstet..

[18] Lynch H. T., Frichot B.C., Lynch J.F. (1978). Familial atypical multiple mole-melanoma syndrome. J. Med. Genet..

[19] Real F.X., Cibrián-Uhalte E., Martinelli P. (2008). Pancreatic cancer development and progression: Remodeling the model. Gastroenterology.

[20] Hruban R.H., Maitra A., Kern S.E., Goggins M. (2007). Precursors to pancreatic cancer. Gastroenterol. Clin. North Am..

[21] Hruban R.H., Maitra A., Goggins M. (2008). Update on pancreatic intraepithelial neoplasia. Int. J. Clin. Exp. Pathol..

[22] Hruban R.H., Maitra A., Schulick R., Laheru D., Herman J., Kern S.E., Goggins M. (2008). Emerging molecular biology of pancreatic cancer. Gastrointest. Cancer Res..

[23] Sipos B., Frank S., Gress T., Hahn S., Klöppel G. (2009). Pancreatic intraepithelial neoplasia revisited and updated. Pancreatology.

[24] Sparr J.A., Bandipalliam P., Redston M.S., Syngal S. (2009). Intraductal papillary mucinous neoplasm of the pancreas with loss of mismatch repair in a patient with Lynch syndrome. Am. J. Surg. Pathol..

[25] Lynch H.T., Voorhees G.J., Lanspa S.J., McGreevy P.S., Lynch J.F. (1985). Pancreatic carcinoma and hereditary nonpolyposis colorectal cancer: a family study. Br. J. Cancer..

[26] Lynch H.T., Fitzsimmons M.L., Smyrk T.C., Lanspa S.J., Watson P., McClellan J., Lynch J.F. (1990). Familial pancreatic cancer: clinicopathologic study of 18 nuclear families. Am. J. Gastroenterol..

[27] Lynch H.T., Fusaro L., Smyrk T.C., Watson P., Lanspa S.J., Lynch J.F. (1995). Medical genetic study of eight pancreatic cancer-prone families. Cancer Invest..

[28] Landi S. (2009). Genetic predisposition and environmental risk factors to pancreatic cancer: A review of the literature. Mutat. Res..

[29] Kastrinos F., Mukherjee B., Tayob N., Wang F., Sparr J., Raymond V.M., Bandipalliam P., Stoffel E.M., Gruber S.B., Syngal S. (2009). Risk of pancreatic cancer in families with Lynch syndrome. JAMA.

[30] Jones S., Zhang X., Parsons D.W., Lin J.C.-H., Leary R.J., Angenendt P., Mankoo P., Carter H., Kamiyama H., Jimeno A., Hong S.-M., Fu B., Lin M.-T., Calhoun E.S., Kamiyama M., Walter K., Nikolskaya T., Nikolsky Y., Hartigan J., Smith D.R., Hidalgo M., Leach S.D., Klein A.P., Jaffee E.M., Goggins M., Maitra A., Iacobuzio-Donahue C., Eshleman J.R., Kern S.E., Hruban R.H., Karchin R., Papadopoulos N., Parmigiani G., Vogelstein B., Velculescu V.E., Kinzler K.W. (2008). Core signaling pathways in human pancreatic cancers revealed by global genomic analyses. Science.

[31] Wang L., Brune K.A., Visvanathan K., Laheru D., Herman J., Wolfgang C., Schulick R., Cameron J.L., Goggins M., Hruban R.H., Klein A.P. (2009). Elevated cancer mortality in the relatives of patients with pancreatic cancer. Cancer Epidemiol. Biomark. Prev..

[32] Al-Sukhni W., Rothenmund H., Borgida A.E., Zogopoulos G., O'Shea A.-M., Pollett A., Gallinger S. (2008). Germline BRCA1 mutations predispose to pancreatic adenocarcinoma. Hum. Genet..

[33] Lynch H.T., Deters C.A., Snyder C.L., Lynch J.F., Villeneuve P., Silberstein J., Martin H., Narod S.A., Brand R.E. (2005). *BRCA1* and pancreatic cancer: pedigree findings and their causal relationships. Cancer Genet. Cytogenet..

[34] Couch F.J., Wang X., McWilliams R.R., Bamlet W.R., de Andrade M., Petersen G.M. (2009). Association of breast cancer susceptibility variants with risk of pancreatic cancer. Cancer Epidemiol. Biomark. Prev..

[35] Hiripi E., Bermejo L., Li X., Sundquist J., Hemminki K. (2009). Familial association of pancreatic cancer with other malignancies in Swedish families. Br. J. Cancer..

[36] Lynch H.T., Krush A.J. (1968). Heredity and malignant melanoma: implications for early cancer detection. Can. Med. Assoc. J..

[37] Lynch H.T., Fusaro R.M., Kimberling W.J., Lynch J.F., Danes B.S. (1983). Familial atypical multiple mole-melanoma (FAMMM) syndrome: segregation analysis. J. Med. Genet..

[38] Lynch H.T., Brand R.E., Hogg D., Deters C.A., Fusaro R.M., Lynch J.F., Liu L., Knezetic J., Lassam N.J., Goggins M., Kern S. (2002). Phenotypic variation in eight extended *CDKN2A* germline mutation familial atypical multiple mole melanoma-pancreatic carcinoma-prone families: the familial atypical multiple mole melanoma-pancreatic carcinoma syndrome. Cancer.

[39] Gargiulo S., Torrini M., Ollila S., Nasti S., Pastorino L., Cusano R., Bonelli L., Battistuzzi L., Mastracci L., Bruno W., Savarino V., Sciallero S., Borgonovo G., Nyström M., Bianchi-Scarrà G., Mareni C., Ghiorzo P. (2009). Germline MLH1 and MSH2 mutations in Italian pancreatic cancer patients with suspected Lynch syndrome. Fam. Cancer..

[40] Pogue-Geile K.L., Chen R., Bronner M.P., Crnogorac-Jurcevic T., White Moyes K., Dowen S., Otey C.A., Crispin D.A., George R.D., Whitcomb D.C., Brentnall T.A. (2006). *Palladin* mutation causes familial pancreatic cancer and suggests a new cancer mechanism. PLoS Med..

[41] Klein A.P., Borges M., Griffith M., Brune K., Hong S.-M., Omura N., Hruban R.H., Goggins M. (2009). Absence of deleterious palladin mutations in patients with familial pancreatic cancer. Cancer Epidemiol. Biomarkers Prev..

[42] Salaria S.N., Illei P., Sharma R., Walter K.M., Klein A.P., Eshleman J.R., Maitra A., Schulick R., Winter J., Ouellette M.M., Goggins M., Hruban R. (2007). Palladin is overexpressed in the non-neoplastic stroma of infiltrating ductal adenocarcinomas of the pancreas, but is only rarely overexpressed in neoplastic cells. Cancer Biol. Ther..

[43] Goicoechea S. M., Bednarski B., Stack C., Cowan D.W., Volmar K., Thorne L., Cukierman E., Rustgi A.K., Brentnall T., Hwang R.F., McCulloch C.A.G., Yeh J.J., Bentrem D.J., Hochwald S.N., Hingorani S.R., Kim H.J., Otey C.A. (2010). Isoform-specific upregulation of palladin in human and murine pancreas tumors. PLoS ONE..

[44] Jones S., Hruban R.H., Kamiyama M., Borges M., Zhang X., Parsons D.W., Lin J.C., Palmisano E., Brune K., Jaffee E.M., Iacobuzio-Donahue C.A., Maitra A., Parmigiani G., Kern S.E., Velculescu V.E., Kinzler K.W., Vogelstein B., Eshleman J.R., Goggins M., Klein A.P. (2009). Exomic sequencing identifies PALB2 as a pancreatic cancer susceptibiliity gene. Science.

[45] Slater E.P., Langer P., Niemczyk E., Strauch K., Butler J., Habbe N., Neoptolemos J.P., Greenhalf W., Bartsch D.K. (2010). PALB2 mutations in European familial pancreatic cancer families. Clin. Genet..

[46] Li A., Omura N., Hong S.-M., Vincent A., Walter K., Griffith M., Borges M., Goggins M. (2010). Pancreatic cancers epigenetically silence *SIP1* and hypomethylate and overexpress *miR-200a/200b* in association with elevated circulating *miR-200a* and *miR-200b* levels. Cancer Res..

[47] Wang W., Chen S., Brune K.A., Hruban R.H., Parmigiani G., Klein A.P. (2007). PancPRO: Risk assessment for individuals with a family history of pancreatic cancer. J. Clin. Oncol..

[48] Parmigiani G., Berry D.A., Aguilar O. (1998). Determining carrier probabilities for breast cancer-susceptibility genes BRCA1 and BRCA2. Am. J. Hum. Genet..

[49] Kim J.E., Lee K.T., Lee J.K., Paik S.W., Rhee J.C., Choi K.W. (2004). Clinical usefulness of carbohydrate antigen 19-9 as a screening test for pancreatic cancer in an asymptomatic population. J. Gastroenterol. Hepatol..

[50] Märten A., Büchler M.W., Werft W., Wente M.N., Kirschfink M., Schmidt J. (2010). Soluble iC3b as an early marker for pancreatic adenocarcinoma is superior to CA19.9 and radiology. J. Immunother. (Hagerstown, Md.: 1997)..

[51] Decker G.A., Batheja M.J., Collins J.M., Silva A.C., Mekeel K.L., Moss A.A., Nguyen C.C., Lake D.F., Miller L.J. (2010). Risk factors for pancreatic adenocarcinoma and prospects for screening. Gastroenterol. Hepatol. (NY).

[52] Yang L., Mao H., Cao Z., Wang Y.A., Peng X., Wang X., Sajja H.K., Wang L., Duan H., Ni C., Staley C.A., Wood W.C., Gao X., Nie S. (2009). Molecular imaging of pancreatic cancer in an animal model using targeted multifunctional nanoparticles. Gastroenterology.

[53] Topazian M., Enders F., Kimmey M., Brand R., Chak A., Clain J., Cunningham J., Eloubeidi M., Gerdes H., Gress F., Jagannath S., Kantsevoy S., LeBlanc J.K., Levy M., Lightdale C., Romagnuolo J., Saltzman J.R., Savides T., Wiersema M., Woodward T., Petersen G., Canto M. (2007). Interobserver agreement for EUS findings in familial pancreatic-cancer kindreds. Gastrointest. Endosc..

[54] Kimmey M.B., Bronner M.P., Byrd D.R., Brentnall T.A. (2002). Endoscopic ultrasound screening for familial pancreatic cancer. Gastrointest. Endosc..

[55] Canto M.I., Goggins M., Hruban R.H., Petersen G.M., Giardiello F.M., Yeo C., Fishman E.K., Brune K., Axilbund J., Griffin C., Ali S., Richman J., Jagannath S., Kantsevoy S.V., Kalloo A.N. (2006). Screening for early pancreatic neoplasia in high-risk individuals: a prospective controlled study. Clin. Gastroenterol. Hepatol..

[56] Kluijt I., Cats A., Fockens P., Nio Y., Gouma D.J., Bruno M.J. (2009). Atypical presentation of FAMMM syndrome with a high incidence of pancreatic cancer: case finding of asymptomatic individuals by EUS surveillance. J. Clin. Gastroenterol..

[57] Yan L., McFaul C., Howes N., Leslie J., Lancaster G., Wong T., Threadgold J., Evans J., Gilmore I., Smart H., Lombard M., Neoptolemos J., Greenhalf W. (2005). Molecular analysis to detect pancreatic ductal adenocarcinoma in high-risk groups. Gastroenterology.

[58] Nakashima A., Murakami Y., Uemura K., Hayashidani Y., Sudo T., Hashimoto Y., Ohge H., Oda M., Sueda T., Hiyama E. (2009). Usefulness of human telomerase reverse transcriptase in pancreatic juice as a biomarker of pancreatic malignancy. Pancreas..

[59] Zhang L., Farrell J.J., Zhou H., Elashoff D., Akin D., Park N.H., Chia D., Wong D.T. (2010). Salivary transcriptomic biomarkers for detection of resectable pancreatic cancer. Gastroenterology.

[60] Cress R.D., Yin D., Clarke L., Bold R., Holly E.A. (2006). Survival among patients with adenocarcinoma of the pancreas: a population-based study (United States). Cancer Causes Contr..

[61] Cameron J.L., Tiall T.S., Coleman J., Belcher K.A. (2006). One thousand consecutive pancreaticoduodenenectomies. Ann. Surg..

[62] Evans D.B., Varadhachary G.R., Crane C.H., Sun C.C., Lee J.E., Pisters P.W.T., Vauthey J.-N., Wang H., Cleary K.R., Staerkel G.A., Charnsangavej C., Lano E.A., Ho L., Lenzi R., Abbruzzese J.L., Wolff R.A. (2008). Preoperative gemcitabine-based chemoradiation for patients with resectable adenocarcinoma of the pancreatic head. J. Clin. Oncol..

[63] Varadhachary G.R., Wolff R.A., Crane C.H., Sun C.C., Lee J.E., Pisters P.W.T., Vauthey J.-N., Abdalla E., Wang H., Staerkel G.A., Lee J.H., Ross W.A., Tamm E.P., Bhosale P.R., Krishnan S., Das P., Ho L., Xiong H., Abbruzzese J.L., Evans D.B. (2008). Preoperative gemcitabine and cisplatin followed by gemcitabine-based chemoradiation for resectable adenocarcinoma of the pancreatic head. J. Clin. Oncol..

